# Outlining a Population “at Risk” of Parkinson's Disease: Evidence from a Case-Control Study

**DOI:** 10.1155/2016/9646057

**Published:** 2016-08-29

**Authors:** Tommaso Schirinzi, Giuseppina Martella, Alessio D'Elia, Giulia Di Lazzaro, Paola Imbriani, Graziella Madeo, Leonardo Monaco, Marta Maltese, Antonio Pisani

**Affiliations:** ^1^Neurology, Department of Systems Medicine, University of Rome Tor Vergata, Via Montpellier 1, 00133 Rome, Italy; ^2^IRCCS Fondazione Santa Lucia, Via del Fosso di Fiorano, Rome, Italy; ^3^Psychiatry, Department of Systems Medicine, University of Rome Tor Vergata, Via Montpellier 1, 00133 Rome, Italy

## Abstract

The multifactorial pathogenesis of Parkinson's Disease (PD) requires a careful identification of populations “at risk” of developing the disease. In this case-control study we analyzed a large Italian population, in an attempt to outline general criteria to define a population “at risk” of PD. We enrolled 300 PD patients and 300 controls, gender and age matched, from the same urban geographical area. All subjects were interviewed on demographics, family history of PD, occupational and environmental toxicants exposure, smoking status, and alcohol consumption. A sample of 65 patients and 65 controls also underwent serum dosing of iron, copper, mercury, and manganese by means of Inductively Coupled-Plasma-Mass-Spectrometry (ICP-MS). Positive family history, toxicants exposure, non-current-smoker, and alcohol nonconsumer status occurred as significant risk factors in our population. The number of concurring risk factors overlapping in the same subject impressively increased the overall risk. No significant differences were measured in the metal serum levels. Our findings indicate that combination of three to four concurrent PD-risk factors defines a condition “at risk” of PD. A simple stratification, based on these questionnaires, might be of help in identifying subjects suitable for neuroprotective strategies.

## 1. Introduction

Parkinson's Disease (PD) is a common neurodegenerative disorder with progressive disabling motor and nonmotor features. A number of therapeutic interventions allow symptomatic relief, but none of them is able to prevent or halt neurodegeneration. In the recent past, through a better comprehension of PD pathogenesis, several molecular pathways have been identified as potential targets of neuroprotection. Unfortunately, clinical trials often failed in translating the encouraging results obtained from* in vitro* and* in vivo* experimental findings. To some extent, the suboptimal selection of enrolled patients and the lack of measurable biomarkers or reliable outcomes account for such failures [1–3]. Genetically defined populations (LRRK2 or GBA mutations) seem to be suitable candidates for neuroprotection [2], but it is well known that less than 10% of PD cases can be ascribed to a monogenic mutation [4]. It is now widely accepted that PD is an idiopathic, multifactorial disease, originating from the interaction between one or more susceptibility loci of the host and one or several environmental modifiers [5–8]. Since numerous PD-risk factors, including positive family history, toxicants exposure, and personal habits, have been identified [5, 6, 9], specific efforts should be made to further define the PD-risk status and, consequently, improve inclusion criteria for neuroprotective treatments. In this case-control study, we screened a large urban population from Italy to outline a population “at risk” of PD. Specifically, we examined the association between PD and risk factors, measured either as single items or in combination. In addition, in support to this PD-risk stratification, we measured serum levels of iron, copper, mercury, and manganese.

## 2. Methods

### 2.1. Study Population

We enrolled 300 consecutive PD patients afferent to* Centro di Riferimento Regionale per il Parkinson della Clinica Neurologica dell'Università “Tor Vergata,”* Rome, Italy, between 2012 and 2015. PD was diagnosed according to UK-PDSBB diagnostic criteria. Enrolled controls were age (±5 years) and sex matched non-blood relatives or friends of patients, not showing signs of parkinsonism or other extrapyramidal signs. Every participant came from the same geographical area (Rome or other cities of Lazio, Italy) and signed an informed consent. All subjects underwent a structured interview. Data collected regarded the following: (1) demographics (name, sex, birth, age at the interview, place of birth, and place of residence); (2) family history of PD (positive = at least one relative of first or second degree affected; negative); (3) occupational exposure (subjects declared the jobs they carried out, years of employment, and use or exposure to three types of toxicants: pesticides/herbicides, chemicals (cleaners, printing products, asbestos, paints, oils, glues, and others), and metals (lead, mercury, manganese, cadmium, chromium, nickel, iron, and copper); to facilitate the report of occupational exposure a list of jobs with the correlating risk of metals exposure has been provided (e.g., painter: lead, manganese, cadmium, etc.)); (4) Environmental exposure (subjects declared the distance between their living place and potential pesticides/herbicides or pollutants sources, toxicants use/exposure for leisure or hobbies; both occupational and environmental exposure were considered as categorical variables (exposed: ≥10 years of exposure); indeed, because of the nature of the data collection (self-reported, retrospective), it was not possible to quantify the exposure precisely [10]); (5) personal habits (subjects declared the smoking status (according the WHO definitions: never-smoker, former-smoker, and current-smoker [11]), the alcohol consumption (no consumption, up to 200 mL/day, and between 200 and 500 mL/day)).

Then, subjects were classified depending on single variables. (1) Family history of PD: it is positive or negative. (2) Toxicants exposure: it includes exposed or nonexposed (exposed = all subjects reporting at least 10-year history of exposure to pesticides/herbicides, chemicals, and metals). According to the cause of exposure, toxicants exposure has been classified into, occupational and environmental. We thus grouped (A) subjects with neither family history of PD or toxicants exposure; (B) subjects with only positive family history of PD; (C) subjects with only toxicants exposure; (D) subjects with “double hit” (positive family history + toxicants exposure). (3) Jobs have been organized into six main categories according to the work setting: agriculture, industry, construction, office, mechanical workers, and other. (4) Smoking status has been divided into current-smoker or non-current-smoker (never-smoker + former-smoker). (5) Alcohol consumption has been divided into nonconsumer and consumer. To avoid or limit any bias, the interviews were conducted by personnel unaware of case status, whereas data were analyzed blindly by distinct operators.

### 2.2. Biochemical Measurements

Compelling evidence demonstrated the role of iron, copper, manganese, and mercury in the pathogenesis of PD [12–17] and a number of studies showed abnormal metal serum levels in PD patients [18, 19]. Since both the environmental pollution and the eating habits may affect the metal concentrations [16, 20–23], such levels could be measured as an index of toxicants exposure. Here we explored serum levels of iron, copper, manganese, and mercury aimed at identifying further elements supporting the PD-risk stratification. We thus selected 65 PD patients and 65 controls, with similar gender and age distributions; normal weight; no history of blood, lung, liver, kidney, or bowel diseases; no previous/ongoing chemotherapy. From each subject we obtained a blood sample in standardized conditions (between 8 and 10 AM, after an overnight fast). Blood was collected in sodium-heparin tubes and centrifuged for 20 min at 2000 rpm at room temperature. After centrifugation, plasma samples were collected and stored at −80°C until analysis. Hemolyzed samples were excluded from the study. Metals serum levels were measured by Farmlab Srl (Guidonia Montecelio, Rome, Italy) through Inductively Coupled-Plasma-Mass-Spectrometry (ICP-MS) [24]. (The study was approved by the local Ethic Committee, number 98-09. All participants signed an informed consent.)

### 2.3. Statistical Analysis

Chi-square test was used to examine differences between groups in categorical variables. Binomial logistic regression was used to calculate the odds ratio (OR) and 95% confidence interval (CI) for the association between PD and each considered variable.

Regarding biochemical measurements, Shapiro-Wilk (W) test demonstrated that the distribution of metal serum levels could not be accurately modeled by normal distribution. Thus, the Mann-Whitney *U* test was used to examine the distribution of serum concentrations between the two groups. Sensitivity and specificity of each metal as biomarker of PD were determined by the receiver operating characteristic (ROC) curve analysis, calculating the area under the curve (AUC). Statistical significance was set at *p* < 0.05. Statistical analysis was performed by IBM SPSS Statistics 22.

## 3. Results

### 3.1. Demographics

PD patients and controls were similar on demographic characteristics, consistent with the matched study design (all the results are summarized in Table 1).

### 3.2. Risk Factors

**Figure 1:**
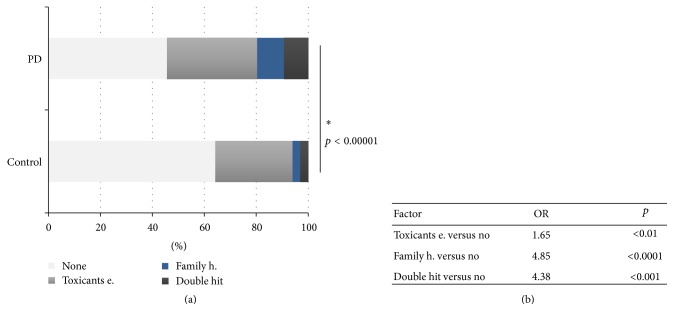
(a) Distribution of risk factors in the two groups (no factors, toxicants exposure, positive family history, “double hit,” or positive family history + toxicants exposure). (b) OR of these risk factors. *∗* means statistical significance.

PD patients showed significantly higher positive family history for PD and percentages of exposed subjects (details in Table 1). The distribution of A, B, C, and D categories (see Section 2) into the two groups was significantly different. Specifically PD patients have greater percentage of B, C, and D categories (PD: none = 45.67%, toxicant exposure 34.67%, positive family history 10.33%, and “double hit” 9.33%; controls: none = 64.3%, toxicant exposure 29.7%, positive family history 3%, and “double hit” 3%; *p* < 0.00001). Among these conditions, positive family history and the “double hit” represent severe risk factors for PD (positive family history OR 4.852, 95% CI 2.238–10.519, and *p* < 0.0001; “double hit” OR 4.383, 95% CI 2.005–9.583, and *p* < 0.0001), whereas the only toxicants exposure is a milder risk factor (OR 1.646, 95% CI 1.151–2,354, and *p* < 0.05) (Figure 1).

### 3.3. External Risk Factors

**Figure 2:**
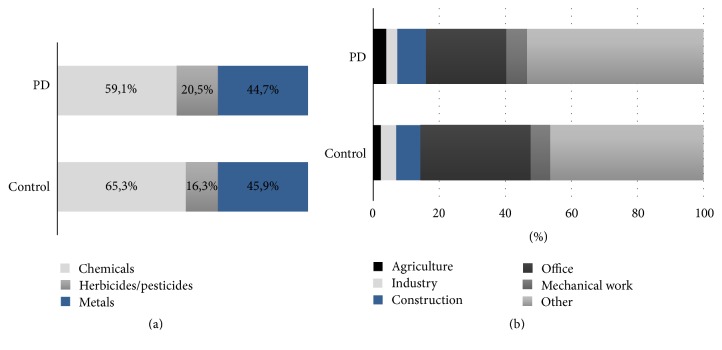
(a) Distribution of types of toxicants handled by the two groups. (b) Distribution of occupational categories in the two groups.

We found, in both cases and controls, occupational exposure as main cause of toxicants exposure (PD: occupational = 77.3%, environmental = 22.7%; controls: occupational 81.6%; environmental = 18.4%; *p* = n.s.). Since the number of persons with occupational exposure was significantly higher in the PD group (PD = 34.3%; controls = 26.7%; *p* < 0.05), this condition represents a PD-risk factor (OR 1.438, 95% CI 1.014–2.040; *p* < 0.05). Regarding toxicant substances, we observed a higher exposure to chemicals and, successively, to metals and herbicides/pesticides, although either PD patients or controls had a multiple exposure (PD: chemicals = 59.1%, metals = 44.7%, and herbicides/pesticides = 20.5%; controls: chemicals = 65.3%, metals = 16.3%, and herbicides/pesticides = 45.9%; *p* = n.s.) (Figure 2(a)). The distribution of job categories was roughly the same between the two groups (PD: agriculture = 4%, industry = 3.33%, construction = 8.67%, office = 24.33%, mechanical workers = 6.33%, and other = 53.33%; controls: agriculture = 2.33%, industry = 4.67%, construction: 7.33%, office = 33.33%, mechanical workers = 6%, and other = 46.33%; *p* = n.s.) (Figure 2(b)). None of these job categories was associated with an increased risk of PD; however some jobs had a higher risk of toxicants exposure (construction: OR 4.549, 95% CI 2.378–8.704, and *p* < 0.0001; mechanical workers: OR 4.199, 95% CI 2.047–8.615, and *p* < 0.0001; agriculture: OR 3.9, 95% CI 1.487–10.227, and *p* < 0.05; industry: OR 3.791, 95% CI 1.6–8.981, and *p* < 0.05).

### 3.4. Personal Habits

**Figure 3:**
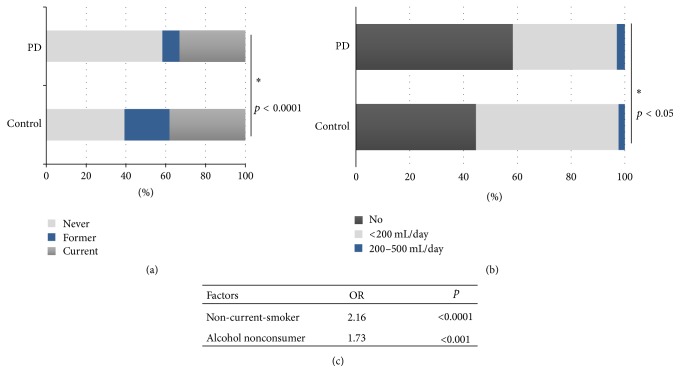
(a) Smoking status in the two groups. (b) Alcohol consumption in the two groups. (c) OR of non-current-smokers and alcohol nonconsumer. *∗* means statistical significance.

PD patients and controls showed different personal habits. The percentages of never-smokers, former-smokers, and current-smokers were different between the groups (*p* < 0.00001) (data in Table 1, Figure 3(a)). Compared to current-smokers, never-smokers (OR 3.88, 95% CI 2.33–6.45, and *p* < 0.0001) and former-smokers (OR 2.27, 95% CI 1.34–3.84, and *p* < 0.05) resulted in having an increased risk of PD. Based on these data, the condition of non-current-smoker may, thus, represent a risk factor for PD (OR 2.16, 95% CI 1.56–2.99, and *p* < 0.0001) (Figure 3(c)). Also in alcohol consumption PD patients and controls behave differently (*p* < 0.05, data in Table 1, Figure 3(b)). We calculated that consuming alcohol may have a protective effect on PD onset (OR 0.65, 95% CI 0.48–0.87, and *p* < 0.05). Therefore, the condition of nonconsumer could imply a risk for PD (OR 1.73, 95% CI 1.26–2.39, and *p* < 0.001) (Figure 3(c)).

### 3.5. Risk Combination

**Figure 4:**
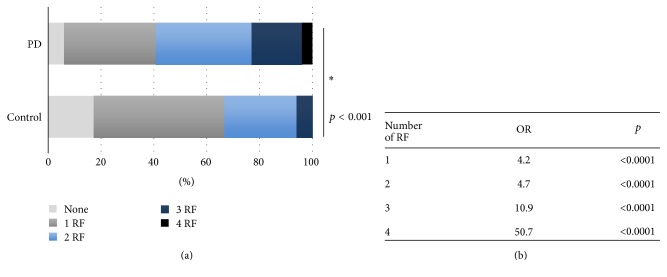
(a) Distribution of the number of concurrent risk factors (RF) in the two groups. (b) OR calculated for number of concurrent risk factors. *∗* means statistical significance.

We identified four solid risk factors for PD (positive family history, toxicants exposure, non-current-smoker status, nonconsumer of alcohol) that can be variably expressed, singularly or in association, in each individual. Therefore, we calculated the number of risk factors present in each subject. In the PD group we observed 6% with no risk factors, 34.7% exhibiting one risk factor, 36.3% presenting two risk factors, 19% with three risk factors, and 4% with four risk factors. Conversely, in controls 17.3% had no risk factor, 49.3% had one risk factor, 27.3% had two risk factors, 6% had three risk factors, and none exhibited all four risk factors (*p* < 0.001) (Figure 4(a)). Binomial logistic regression demonstrated that the number of concurrent risk factors significantly predicts PD. Indeed, we measured that the presence of one factor has OR 4.22, 95% CI 2.36–7.55, and *p* < 0.00001; two factors OR 4.75, 95% CI 2.65–8.49, and *p* < 0.00001; three factors OR 10.94, 95% CI 5.37–22.28, and *p* < 0.00001; four factors OR 50.67, 95% CI 6.18–415.29, and *p* < 0.00001 (Figure 4(b)). These findings thus suggest that combination of three to four risk factors determines a statistically significant increase in the risk of developing PD.

### 3.6. Metal Serum Levels

By using the ICP-MS, we measured in the PD group a mean ± SD concentration of 926.04 ± 444.91 microg/L for iron, of 981.32 ± 273.56 microg/L for copper, of 28 ± 21 microg/L for mercury, and of 3.43 ± 1.77 microg/L for manganese. Instead, in the control group we detected a mean ± SD concentration of 965.39 ± 627.54 microg/L for iron, of 1061.11 ± 423.77 microg/L for copper, of 48 ± 33 microg/L for mercury, and of 1.78 ± 0.71 microg/L for manganese. Although the mean manganese concentration was higher in PD, the distribution of all the values was not normal. The Mann-Whitney *U* test excluded significant differences in the metals serum levels between the two groups and, accordingly, the ROC curve analysis failed to provide significant results. Therefore, in our population we did not observe relevant differences in the blood levels of iron, copper, mercury, and manganese excluding their function as toxicants exposure indexes or PD-risk factors. Indeed, it is likely that a remote exposure does not change the present blood concentration of metals. However, it should be reminded that, because of either the biological variability of the elements or the variety of biochemical assays, the scientific literature did not yet provide univocal data on metals serum concentrations in PD [18].

## 4. Discussion

The results of this case-control study allow a clear and consistent PD-risk stratification based on pure anamnestic information obtained by counting the number of concurrent PD-risk factors.

As first step, we screened a large urban Italian population for few PD-risk factors. In agreement with previous reports, we confirmed the importance of a positive family history (OR 4.852) in the pathogenesis of PD [5, 6, 9]. Also the toxicants exposure (OR 1.646) has proven being PD-risk factor [9, 25], particularly when it occurs in subjects with a familiar predisposition (OR 4.383), supporting the well-established “double hit” pathogenic hypothesis [5, 26, 27]. In our population, the occupational exposure exceeds environmental exposure and represents a significant risk factor (OR 1.438). Although some professional categories have been historically considered at risk of PD (farmer, welder) [9, 25, 28], in our study we did not find a particularly dangerous occupation. However, we noticed that people working in constructions and mechanicals handle more frequently toxicants, which essentially consist in chemical products or metals. Regarding environmental exposure, in our geographical area the air pollution is a relevant matter [29] which acts as a source of multiple toxicants, including metals and chemicals, implicated in triggering the neurodegenerative diseases [30, 31]. Unfortunately, here we did not examine the eating habits and the taking of industrially derived foods, which may similarly contain traces of toxicants or abnormal concentrations of metals and minerals. However, since both the environmental pollution and nutrition can modify physiological body levels of metals [16, 20–23], we measured serum concentration of iron, copper, mercury, and manganese as indexes of environmental toxicants exposure, but we did not find significant differences between PD and controls.

The personal habits, such as smoking status and alcohol consumption, have been extensively studied in relation to the PD-risk. Specifically, regarding smoking, it has been demonstrated either that smokers have a lower prevalence of PD or that quitting smoking could represent a potential early nonmotor feature of PD [6, 9, 11, 32–34]. The evaluation of the smoking status in our population measured a consistent PD-risk for the non-current-smokers (OR 2.16). Instead, with regard to the alcohol consumption, several meta-analyses, despite many confounding factors, have reported an inverse correlation between alcohol intake and PD-risk [25, 35, 36]. Also in our case, we observed that PD patients are less prone to alcohol consumption, and coherently the nonconsumers have a significant PD-risk (OR 1.73).

Each of these four risk factors (positive family history, toxicants exposure, non-current-smoker, alcohol nonconsumer) has its own “risk power,” the greatest belonging to the positive family history. However, many factors frequently overlap in the same person, increasing the PD-risk in direct proportion to their number. Precisely, we reported that in the presence of three or four of the mentioned risk factors, subjects are highly “at risk” of PD (resp., OR 10.94, OR 50.67). Our data thus suggest that it is possible to stratify the PD-risk by means of the recognition of the concurrent risk factors, allowing an early identification of populations “at risk” of PD. Currently, the adopted inclusion criteria for PD neuroprotective trials are idiopathic RBD (REM Behavior Disorder) and anosmia, which could both precede other neurodegenerative diseases and already underlie a neurodegeneration, inducing erroneous patients selection. [37–41]. Conversely, we profiled a simple PD-risk stratification, applicable to the general population, in order to select a group suitable for PD “primary prevention,” as it has been commonly done with cardiovascular diseases. In fact, primary prevention in PD could be directed towards the external modifiable risk factors, as occupational toxicants exposure or environmental pollutants. Alternatively, other behaviors inversely associated with PD-risk and thus useful as primary preventive strategies are physical exercise [42], drinking green tea [43], and eating vitamins-rich aliment [16].

## 5. Conclusions

Simple and early stratification of PD-risk, helpful in identifying subjects suitable for neuroprotective strategies, can be achieved by counting the number of the main PD-risk factors (positive family history, toxicants exposure, non-current-smoker, and alcohol nonconsumer).

## Figures and Tables

**Table 1:** Demographics and main risk factors in our study population.

Variable	Group			*p*
	Total *n* = 600	PD *n* = 300	Control *n* = 300	
*Sex*				ns
Male (%)	50.5	51	50	
Female (%)	49.5	49	50	

*Age at interview *				ns
Mean ± st. dev.	72.8 ± 10.3	70.6 ± 10.4	69.4 ± 9.4	
40–70 y (%)	49.7	47	53	
71–100 y (%)	50.3	53	47	

*Family history of PD*				<0.000001
Negative (%)	87.2	80.3	94	
Positive (%)	12.8	19.7	6	

*Toxicants exposure*				<0.001
No (%)	62	56	67.3	
Yes (%)	38	44	32.7	

*Smoking status*				<0.00001
Never (%)	48.8	58.3	39.3	
Former (%)	15.7	8.7	22.7	
Current (%)	35.5	33	38	

*Alcohol consumption*				<0.05
No (%)	51.5	58.3	44.7	
<200 mL/day (%)	45.8	38.7	53.0	
200–500 mL/day (%)	2.7	3.0	2.3	

## References

[1] Athauda D., Foltynie T. (2015). The ongoing pursuit of neuroprotective therapies in Parkinson disease. *Nature Reviews Neurology*.

[2] Schapira A. H. V., Olanow C. W., Greenamyre J. T., Bezard E. (2014). Slowing of neurodegeneration in Parkinson's disease and Huntington's disease: future therapeutic perspectives. *The Lancet*.

[3] Stocchi F. (2014). Therapy for Parkinson's disease: what is in the pipeline?. *Neurotherapeutics*.

[4] Trinh J., Farrer M. (2013). Advances in the genetics of Parkinson disease. *Nature Reviews Neurology*.

[5] Kitada T., Tomlinson J. J., Ao H. S., Grimes D. A., Schlossmacher M. G. (2012). Considerations regarding the etiology and future treatment of autosomal recessive versus idiopathic parkinson disease. *Current Treatment Options in Neurology*.

[6] Tanner C. M. (2010). Advances in environmental epidemiology. *Movement Disorders*.

[7] Petrucci S., Consoli F., Valente E. M. (2014). Parkinson disease genetics: a ‘continuum’ from mendelian to multifactorial inheritance. *Current Molecular Medicine*.

[8] De Rosa P., Marini E. S., Gelmetti V., Valente E. M. (2015). Candidate genes for Parkinson disease: lessons from pathogenesis. *Clinica Chimica Acta*.

[9] Noyce A. J., Lees A. J., Schrag A. (2016). The prediagnostic phase of Parkinson's disease. *Journal of Neurology, Neurosurgery & Psychiatry*.

[10] Litvan I., Lees P. S. J., Cunningham C. R. (2016). Environmental and occupational risk factors for progressive supranuclear palsy: Case-Control Study. *Movement Disorders*.

[11] Moccia M., Erro R., Picillo M. (2015). Quitting smoking: an early non-motor feature of Parkinson's disease?. *Parkinsonism and Related Disorders*.

[12] Jan A. T., Azam M., Siddiqui K., Ali A., Choi I., Haq Q. M. R. (2015). Heavy metals and human health: mechanistic insight into toxicity and counter defense system of antioxidants. *International Journal of Molecular Sciences*.

[13] Ward R. J., Zucca F. A., Duyn J. H., Crichton R. R., Zecca L. (2014). The role of iron in brain ageing and neurodegenerative disorders. *The Lancet Neurology*.

[14] Davies K. M., Mercer J. F., Chen N., Double K. L. (2016). Copper dyshomoeostasis in Parkinson's disease: implications for pathogenesis and indications for novel therapeutics. *Clinical Science*.

[15] Bouabid S., Tinakoua A., Lakhdar-Ghazal N., Benazzouz A. (2016). Manganese neurotoxicity: behavioral disorders associated with dysfunctions in the basal ganglia and neurochemical transmission. *Journal of Neurochemistry*.

[16] Agim Z. S., Cannon J. R. (2015). Dietary factors in the etiology of Parkinson's disease. *BioMed Research International*.

[17] Chin-Chan M., Navarro-Yepes J., Quintanilla-Vega B. (2015). Environmental pollutants as risk factors for neurodegenerative disorders: Alzheimer and Parkinson diseases. *Frontiers in Cellular Neuroscience*.

[18] Zhao H. W., Lin J., Wang X. B. (2013). Assessing plasma levels of selenium, copper, iron and zinc in patients of Parkinson's disease. *PLoS ONE*.

[19] Ahmed S. S. S. J., Santosh W. (2010). Metallomic profiling and linkage map analysis of early Parkinson's disease: a new insight to aluminum marker for the possible diagnosis. *PLoS ONE*.

[20] Casjens S., Henry J., Rihs H.-P. (2014). Influence of welding fume on systemic iron status. *Annals of Occupational Hygiene*.

[21] Fukushima T., Tan X., Luo Y., Kanda H. (2010). Relationship between blood levels of heavy metals and Parkinson's disease in China. *Neuroepidemiology*.

[22] Logroscino G., Gao X., Chen H., Wing A., Ascherio A. (2008). Dietary iron intake and risk of Parkinson's disease. *American Journal of Epidemiology*.

[23] Hagemeier J., Tong O., Dwyer M. G., Schweser F., Ramanathan M., Zivadinov R. (2015). Effects of diet on brain iron levels among healthy individuals: an MRI pilot study. *Neurobiology of Aging*.

[24] Konz T., Alonso-García J., Montes-Bayón M., Sanz-Medel A. (2013). Comparison of copper labeling followed by liquid chromatography-inductively coupled plasma mass spectrometry and immunochemical assays for serum hepcidin-25 determination. *Analytica Chimica Acta*.

[25] Noyce A. J., Bestwick J. P., Silveira-Moriyama L. (2012). Meta-analysis of early nonmotor features and risk factors for Parkinson disease. *Annals of Neurology*.

[26] Cannon J. R., Greenamyre J. T. (2013). Gene-environment interactions in Parkinson's disease: specific evidence in humans and mammalian models. *Neurobiology of Disease*.

[27] Martella G., Madeo G., Maltese M. (2016). Exposure to low-dose rotenone precipitates synaptic plasticity alterations in PINK1 heterozygous knockout mice. *Neurobiology of Disease*.

[28] van der Mark M., Vermeulen R., Nijssen P. C. G. (2015). Occupational exposure to solvents, metals and welding fumes and risk of Parkinson's disease. *Parkinsonism and Related Disorders*.

[29] http://www.arpalazio.gov.it/.

[30] Levesque S., Surace M. J., McDonald J., Block M. L. (2011). Air pollution and the brain: subchronic diesel exhaust exposure causes neuroinflammation and elevates early markers of neurodegenerative disease. *Journal of Neuroinflammation*.

[31] Mumaw C. L., Levesque S., McGraw C. (2016). Microglial priming through the lung-brain axis: the role of air pollution-induced circulating factors. *The FASEB Journal*.

[32] Li X., Li W., Liu G., Shen X., Tang Y. (2015). Association between cigarette smoking and Parkinson's disease: a meta-analysis. *Archives of Gerontology and Geriatrics*.

[33] Cicchetti F., Drouin-Ouellet J., Gross R. E. (2009). Environmental toxins and Parkinson's disease: what have we learned from pesticide-induced animal models?. *Trends in Pharmacological Sciences*.

[34] Tanner C. M., Goldman S. M., Aston D. A. (2002). Smoking and Parkinson's disease in twins. *Neurology*.

[35] Ishihara L., Brayne C. (2005). A systematic review of nutritional risk factors of Parkinson's disease. *Nutrition Research Reviews*.

[36] Bettiol S. S., Rose T. C., Hughes C. J., Smith L. A. (2015). Alcohol consumption and Parkinson's disease risk: a review of recent findings. *Journal of Parkinson's Disease*.

[37] Iranzo A., Molinuevo J. L., Santamaría J. (2006). Rapid-eye-movement sleep behaviour disorder as an early marker for a neurodegenerative disorder: a descriptive study. *Lancet Neurology*.

[38] Postuma R. B., Iranzo A., Hogl B. (2015). Risk factors for neurodegeneration in idiopathic rapid eye movement sleep behavior disorder: a multicenter study. *Annals of Neurology*.

[39] Mahlknecht P., Iranzo A., Högl B. (2015). Olfactory dysfunction predicts early transition to a Lewy body disease in idiopathic RBD. *Neurology*.

[40] Postuma R. B., Gagnon J.-F., Bertrand J.-A., Génier Marchand D., Montplaisir J. Y. (2015). Parkinson risk in idiopathic REM sleep behavior disorder: preparing for neuroprotective trials. *Neurology*.

[41] Kim Y. K., Yoon I.-Y., Kim J.-M. (2010). The implication of nigrostriatal dopaminergic degeneration in the pathogenesis of REM sleep behavior disorder. *European Journal of Neurology*.

[42] Paillard T., Rolland Y., de Barreto P. S. (2015). Protective effects of physical exercise in Alzheimer’s disease and Parkinson’s disease: a narrative review. *Journal of Clinical Neurology*.

[43] Caruana M., Vassallo N., Vassallo N. (2015). Tea polyphenols in Parkinson’s disease. *Natural Compounds as Therapeutic Agents for Amyloidogenic Diseases*.

